# Understanding Uncertainties in Non-Linear Population Trajectories: A Bayesian Semi-Parametric Hierarchical Approach to Large-Scale Surveys of Coral Cover

**DOI:** 10.1371/journal.pone.0110968

**Published:** 2014-11-03

**Authors:** Julie Vercelloni, M. Julian Caley, Mohsen Kayal, Samantha Low-Choy, Kerrie Mengersen

**Affiliations:** 1 School of Mathematical Sciences, Queensland University of Technology, Brisbane, Queensland, Australia; 2 Australian Institute of Marine Science, Townsville, Queensland, Australia; 3 Bren School of Environmental Science and Management, University of California Santa Barbara, Santa Barbara, California, United States of America; Southwest University, China

## Abstract

Recently, attempts to improve decision making in species management have focussed on uncertainties associated with modelling temporal fluctuations in populations. Reducing model uncertainty is challenging; while larger samples improve estimation of species trajectories and reduce statistical errors, they typically amplify variability in observed trajectories. In particular, traditional modelling approaches aimed at estimating population trajectories usually do not account well for nonlinearities and uncertainties associated with multi-scale observations characteristic of large spatio-temporal surveys. We present a Bayesian semi-parametric hierarchical model for simultaneously quantifying uncertainties associated with model structure and parameters, and scale-specific variability over time. We estimate uncertainty across a four-tiered spatial hierarchy of coral cover from the Great Barrier Reef. Coral variability is well described; however, our results show that, in the absence of additional model specifications, conclusions regarding coral trajectories become highly uncertain when considering multiple reefs, suggesting that management should focus more at the scale of individual reefs. The approach presented facilitates the description and estimation of population trajectories and associated uncertainties when variability cannot be attributed to specific causes and origins. We argue that our model can unlock value contained in large-scale datasets, provide guidance for understanding sources of uncertainty, and support better informed decision making.

## Introduction

Effective decision making in conservation and management can be hindered by uncertainty surrounding our understanding of population trajectories [1]. Improving our understanding of the variability in temporal fluctuations in species abundances is a particular challenge when using ecological data because it includes both endogenous variation in species dynamics as well as errors of observation and estimation [2]–[5]. This situation is complicated further because the long-term and broad-scale surveys that are needed to efficiently capture ecological trends typically exhibit variability that increases with the scale of observation [6].

There is an acknowledged lack of statistical tools able to efficiently deal with non-linear trajectories, multi-scale observations and the resulting uncertainties in ecology [7]–[9]. This lack of tools is particularly relevant for coral reefs where highly variable species dynamics and complex multi-dimensional interaction pathways make it difficult to construct models that adequately describe both overall (linear) trends and (nonlinear) fluctuations or deviations around these trends, and also sufficiently accommodate uncertainties inherent in these species trajectories [10]–[18]. Ignoring such nonlinearities and uncertainties, however, can lead to imprecise inferences and compromise effective management of ecosystems. For example, differences in model specification have led to disagreements in interpretation of reef trajectory and health [13], [18]–[21].

Globally, coral reefs face diverse threats that are increasing in frequency and intensity, yet the knowledge required to manage them effectively is currently unavailable [22]. These threats are contributing to a global decline of corals, the foundation species in these ecosystems, through direct sources of mortality (e.g. destructive fishing, coastal development, anchorage damage) and degradation of reef environments from different forms of pollution, over-harvesting, and climate change [23]. These pressures can affect corals across spatial scales ranging from a few cm^2^ to thousands of km^2^, and across temporal scales ranging from seconds to decades and centuries [17], [24], [25]. Despite general awareness that corals are being seriously impacted by the simultaneous effects of these multiple sources of degradation, little attention has been given to the importance of spatial scale in models of coral trajectories [9], [13], [26]. Indeed, most models of coral trajectories have focused on a single spatial scale using either the scale of observation, or by aggregating data from multiple sources to achieve some degree of generalization at a larger scale [27]–[29]. Both of these approaches, however, fail to capture the causal mechanisms leading to the actual trajectory observed in nature, and their variability in time and space [18], [30], [31].

Recent multi-scale approaches to understanding coral trajectories have used generalized linear mixed models (GLMMs). These trajectories are typically fitted using linear equations [12], [16], [17] or natural splines [13], [14]. In such cases, coral trends at larger spatial scales (e.g. regions and subregions) are usually fitted by taking into account the hierarchical structure of smaller scales (e.g. habitats, reefs and sites) nested within these larger levels [12], [13], [16], [32]. GLMMs typically accommodate this hierarchical structure in the trends, or fixed components, by including random effects in the parameterization of the model [33]. However, when using such methods, care is required to account realistically for the spatial and/or temporal structure affecting random effects [2]. In all of these studies, random effects are typically assumed to be identically, normally distributed and independently at a particular spatial scale, such as reef [12]–[14], [16]. Since random effects are not directly observed in such an analytical framework, the validity of these assumptions is difficult to test, and if violated, can lead to model misspecifications [2]. Of concern is that violation of these assumptions may be particularly important when investigating coral trajectories. Indeed, corals as well as other sessile habitat-forming communities are often studied using long-term surveys over large spatial scales [11], [13], [27]. Data from such surveys are likely to exhibit temporal and/or spatial structure, with measurements made close together in space and or time being closely correlated. Such correlations are also likely to be reinforced by large-scale disturbances that can affect multiple locations simultaneously and for long periods [12], [16], [34]–[36]. Consequently, commonly used statistical approaches described above, specify spatio-temporal structure in the fixed-effects component, but not in the random effects. Such methods, therefore, are limited in their ability to realistically describe the underlying generative processes and the different sources of uncertainty commonly encountered in ecological monitoring data [4], [26], [37].

An alternative approach to modelling large-scale survey data is to break the overall model into a series of hierarchically organized sub-models and embed them in a Bayesian analytical framework. These sub-models collectively describe a joint probability distribution for parameters of interest and for predictions. By construction, the model also accounts for differing sources of uncertainty emerging at differing stages in the investigation [2], [38], including measurement error in data collection (stage 1), process error in representation of the ecological process(es) of interest (stage 2), and statistical error in the estimation of parameters (stage 3). The flexibility of this formulation, coupled with simulation-based computational methods for parameter estimation such as Markov Chain Monte Carlo and related algorithms, can be harnessed to produce more ecologically sensitive nonlinear models. Using this approach, the posterior predictive distribution, which incorporates all three levels and other relevant prior information, can be used to evaluate the adequacy of the model in terms of its ability to describe the observed data as well as predict unobserved data including future or missing observations [3]. These models have the added advantage of being able to be updated when more data become available, thereby facilitating continual refinement of knowledge about ecological processes and considerably increasing their predictive capability [39].

We introduce a Bayesian semi-parametric hierarchical model to simultaneously quantify nonlinearities in the relationships between a response variable, coral cover, and its covariates, in this case, time, uncertainties associated with the model structure and associated parameters, and error associated with multiple scales of observation. We illustrate this approach using 14 years of population estimates for the major reef-building coral genus *Acropora*, from the northern Great Barrier Reef (GBR), Australia. We provide posterior estimates and associated uncertainties for coral population trajectories among sites-, reefs- and habitats within a sub-region, and within reefs and sites across time. Our purpose is not to show a full analysis of the dataset, but rather to illustrate the modelling concept and its broader application. We demonstrate that this modelling approach is well suited to estimation of population trajectories and corresponding uncertainty in cases where population variability cannot be assigned to unique causes and origins. We argue that this approach provides a useful tool for investigating environmental drivers of population trajectories, the scales at which they act, and which is applicable to a wide range of species. In doing so, it facilitates greater comprehension of the uncertainties associates with trajectories of these species and, thereby supports more informed decision making.

## Methods

### Data

We used estimates of coral cover from the Australian Institute of Marine Science's Long Term Monitoring Program (LTMP) of the GBR [40]. The LTMP sampled benthic communities annually from 1994 to 2004, and then every second year, on 47 reefs throughout the GBR using five permanent 50×1 m^2^ photo- (prior to 2006) and video-transects (from 2006) between 6 and 9 m depth [41]. Hard coral cover was estimated at the genus level and expressed as a percentage, based on estimates taken from 200 random points along each transect (see [41] for further details). In this study, we restrict our investigation to the dynamics of acroporid corals which dominate the GBR (i.e. 51% of the coral cover) and are considered responsible for most of the variability observed in its coral community trajectories [12].

**Figure 1:**
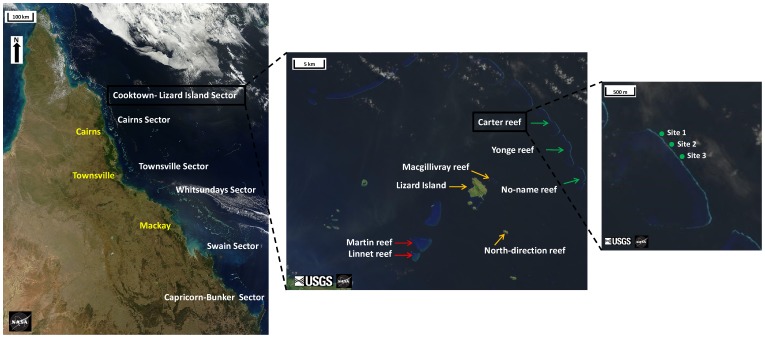
Sampling design of the Long Term Monitoring Program showing the hierarchical levels of observation for the Cooktown-Lizard Island sub-region, one of six sub-regions of Australia's Great Barrier Reef. The sub-region is divided into three shelf-positions or habitats (inner reef (red arrows), mid-shelf reef (orange arrows), and outer reef (green arrows)). Each habitat is sampled on three sites (green dots) in each of two or three reefs. We highlight three sites at Carter reef from the outer reef habitat. Modified from original satellite images © Landsat and MODIS satellite imagery courtesy of NASA Goddard Space Flight Center and US Geological Survey.

To minimize geographical variability in coral dynamics [14], we parameterized the model using data from a single sub-region within the GBR, the Cooktown-Lizard Island section for the period 1994 to 2010 inclusive (Fig. 1). This sub-region is the northern-most section of the GBR sampled by the LTMP, spanning a latitudinal range from 14°S to 15°50'S. Within this area, the LTMP samples three reef habitats defined by their positions on the continental shelf. Inner-reefs are the closest to the coast and are most exposed to terrestrial and human influences [23]. The mid-shelf habitat extends over a large part of the GBR lagoon, with reefs situated at various distances between inner and outer habitats of the barrier reef. The outer-reef habitat extends into more oceanic conditions. In this sub-region, the survey is spatially replicated on two to three reefs per habitat, each reef being itself sampled at three distinct sites (Fig. 1). We investigated variability in coral dynamics at four spatial scales of observation of this hierarchical sampling design ranging from ∼250 m^2^ for the site scale, 1 km^2^ for the reef scale, 5–45 km^2^ for the habitat scale, and ∼500 km^2^ for the sub-regional scale of the Cooktown-Lizard Island section of the GBR. Observations at the transect scale within sites were pooled because there were no significant differences among transects after testing.

### Model

A Bayesian longitudinal semi-parametric regression model was used to assess trends in the study region. Longitudinal models are suitable for situations like this where repeated measurements on several individuals (here sites within reefs) have been made to reflect how a variable of interest behaves through time. Temporal dynamics are often too complex to be modelled parametrically, when the relationship between time and the response variable cannot be assumed to follow a specific and consistent pattern. Such patterns are typical for ecological data from corals and many other natural populations and communities whose dynamics are driven by myriad interacting biotic and abiotic factors [7]. Semi-parametric regressions offer an attractive approach for modelling such data. They combine a linear model and a smooth non-linear function, and therefore, provide a trade-off between flexibility and interpretability of results [42]. In this paper, penalized splines were used to describe the non-linear components of the model [43]. Within- and between-group variability was accounted for by the inclusions of random effects terms and differing levels of spatial hierarchy in the structure of the model. The preferred model was selected based on Deviance Information Criterion (DIC) diagnostics, which take into account both goodness of fit and model parsimony [44].

Let *y_ij_* represent the proportion of *Acropora* spp. observed at time *t_ij_* for a site *i* at the *j^th^* time point. There are a number of potential ways for describing the distribution of *y_ij_*; for example, it could be represented by a Beta distribution (continuous over the range 0 to 1) or by a normal distribution based on an arcsine or angular transformation [45]. Here, we adopt the latter approach, so that the dynamics of *Acropora* are modelled as:
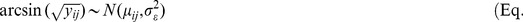
(Eq.\ 1)where the transformed value of *Acropora* coverage is assumed to be normally distributed with an expected value *µ_ij_* and measurement error term σ_ε_. We use a very weakly informative conjugate prior for the variance with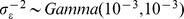.

The expected value *µ_ij_* is modelled as:
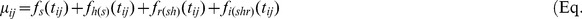
(Eq.\ 2)where *f_s_* (.) describes the overall mean curve at the scale of the sub-region, and deviations from this overall curve represent hierarchically the habitat-, reef-, and site-specific trajectories. These scales are indexed *h*, *r* and *i* respectively with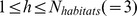, 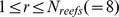 and 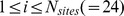. In the following, for clarity and where it does not cause confusion, the nested subscripts are ignored.

All four contributions to the expected response are modelled as combinations of linear trends and splines:
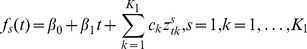


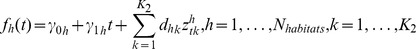


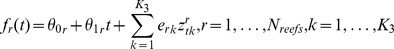


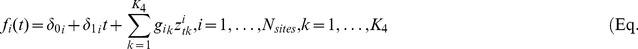
(Eq.\ 3)


The matrix 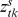 is the (*t, k*)^th^ entry of the design matrix [43], for the penalized spline random coefficients *{c_k_}*, corresponding to the sub-region mean function *f_s_* (⋅). Similarly, 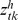 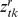, and 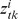 are defined as the (*t, k*)^th^ entries of the design matrices for random coefficients corresponding to habitat level *f_h_* (⋅), the reef level *f_r_* (⋅), and site scale curves *f_i_* (⋅). At the habitat scales one set of random coefficients *{d_hk_}* is allocated to each habitat. Similarly at reef and site scales one set of random coefficients *{e_rk_}* or *{g_ik_}* are allocated to each reef or site, respectively. Four knots were used for the splines for each curve (i.e. *K_1_ = … = K_4_ = 4*). Note that because the smoothing is controlled by a penalty parameter, the number of knots *K* is not a crucial parameter in the model [46]. For modelling trajectories over time *t*, the model allows random slopes and intercepts *θ_r_* and *δ_s_* at the reef- and site-specific scales. Trajectories at the sub-regional and habitat scales are considered as fixed effects, modelled via *β_s_* and *γ_h_*. The time regressor (*t*) is centered on the year 2001 to facilitate model convergence, and to minimize correlation among random effects [17]. Autocorrelated temporal and spatial terms are not explicitly defined in this model, since the correlation exhibited in the data was largely incorporated through the hierarchical structure and the assignment of random effects at each stage of the model [8], [17]. This was checked by plotting residuals at each modelled spatial scale and using the autocorrelation function to confirm an absence of temporal structure in the fitted model (results not shown).

The model also assumes that the *c*, *d*, *e*, *g, θ* and *δ* parameters are mutually independent, with hierarchical priors defined to model the random effects *σ^2^* as follows:
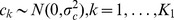


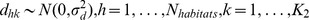


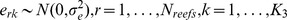


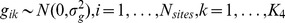


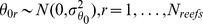


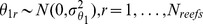


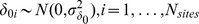


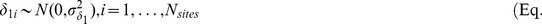
(Eq.\ 4)


The amount of shrinkage induced by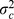,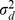,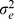, 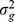 is allowed to differ for each level of the model and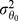,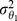,
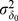
and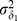 represent variability of intercepts, and slopes among reefs and sites, respectively.

Temporal trends at sub-regional and habitat scales are considered specific to each area, and hence fitted using fixed effects, with vaguely informative prior distributions:
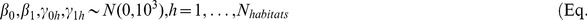
(Eq.\ 5)


We use conjugate Gamma priors on the random effects parameters:
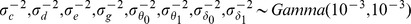


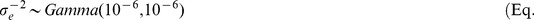
(Eq.\ 6)


Other prior formulations for the precisions were also evaluated, including the Uniform and half-Cauchy distributions for the corresponding standard deviations [47], with no substantive effect on the posterior distributions or inferences (results not shown).

**Figure 2:**
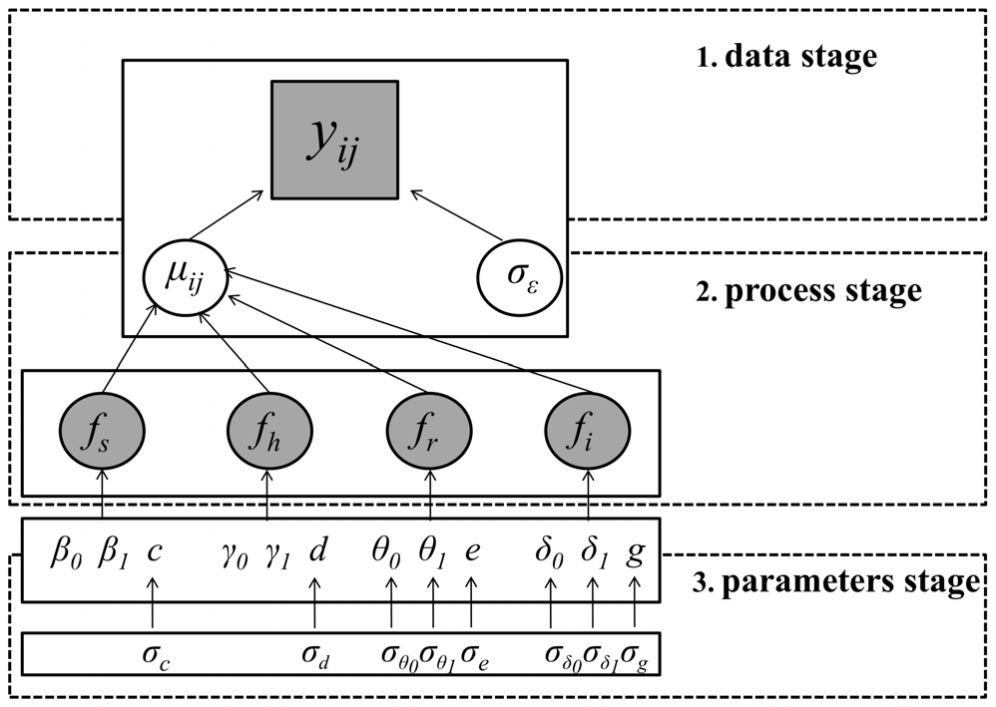
Directed Acyclic Graph showing how *Acropora* cover *y_ij_* at a site *i* and time *j* is fitted in three stages. Here, the transformed data *y_ij_* are modelled using a normal distribution with an expected value *µ_ij_* and variance σ_ε_. The expected value *µ_ij_* is a function of four spatial scales (sub-region *f_s_*, habitat *f_h_*, reef *f_r_*, and site *f_i_*). Linear trends at sub-region and habitat scale are modelled using fixed effects (*β*, *γ*). At the site and reef scale, trend parameters (*θ*, *δ*) are considered as random. Trend parameters are denoted with the subscript 0 corresponding to the intercept and 1 corresponding to the slope. Random effects require specification of a variance component (*σ*), specified for each spatial scale of the model. Refer to the methods section for equations.

The Directed Acyclic Graphic (DAG) presented in Figure 2 shows connections between the three hierarchical stages of the model. At the first stage, the data level, the arcsine transformed value of the response *y_ij_* is distributed around a population mean *µ_ij_* with measurement error σ_ε_ (Eq. 1). The second stage comprises the model for the ecological process and the associated process error (Eq. 2–4). The third stage includes uncertainty in the trend parameters (Eq. 5) and variance components (Eq. 6).

Analysis was performed using the R package R2WinBUGS [48] to call the Bayesian software analysis WinBUGS [49]. We provide R and WinBUGS code in the supporting information file. Posterior distributions of parameters were approximated by Markov Chain Monte Carlo with 200,000 iterations. Convergence diagnostics were assessed by using visual (trace and density-plots of parameters and autocorrelation plots between MCMC draws) and statistical (Gelman and Rubin diagnostic) functions from the R package coda [50]. Convergence was satisfied using a burn-in of 100,000 iterations. Three MCMC chains were simultaneously run to further evaluate and confirm convergence to stationarity. A thinning rate of 50 iterations, mainly used to reduce computer storage space, also improved the independence of the simulated values. Parameter inferences were drawn from posterior distributions constructed from the retained 2000 iterations from the MCMC chains. Goodness-of-fit was assessed by overall model fit diagnostics (DIC), approximate normal distributions for the root-mean-squared error (RMSE) terms, precision of parameter estimates (width of credible intervals) and posterior predictive fit (whether the observed values were contained in the 95% credible intervals obtained from the respective posterior predictive distributions). Autocorrelations between parameters within a spatial scale were also examined to confirm independence between linear slope and intercept terms. The accuracy of model outputs at each spatial scale was assessed by inspecting the posterior distributions of the trend parameters: *γ_0h_*, *γ_1h_*, *θ_0r_*, *θ_1r_*, *δ_0i_* and *δ_1i_*. The fitted trajectory model was then decomposed into linear and non-linear components by splitting the respective equations (Eq. 3) into two parts (for example, linear: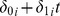 and non-linear:
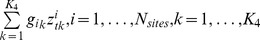
for *f_i_(t)*). This decomposition was visualized for each spatial scale. Note that we examine different components of the expected value *µ_ij_* depending on the spatial scale. Coverage of *Acropora* at the habitat scale is a function of the contribution of the sub-region and habitat 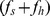
whereas at the reef level it is a function of the contribution of the sub-region, habitat and reef 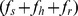. Finally at the site scale we retrieve the expected value of observations 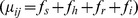.

## Results

### Posterior distributions of model parameters and uncertainty

**Figure 3:**
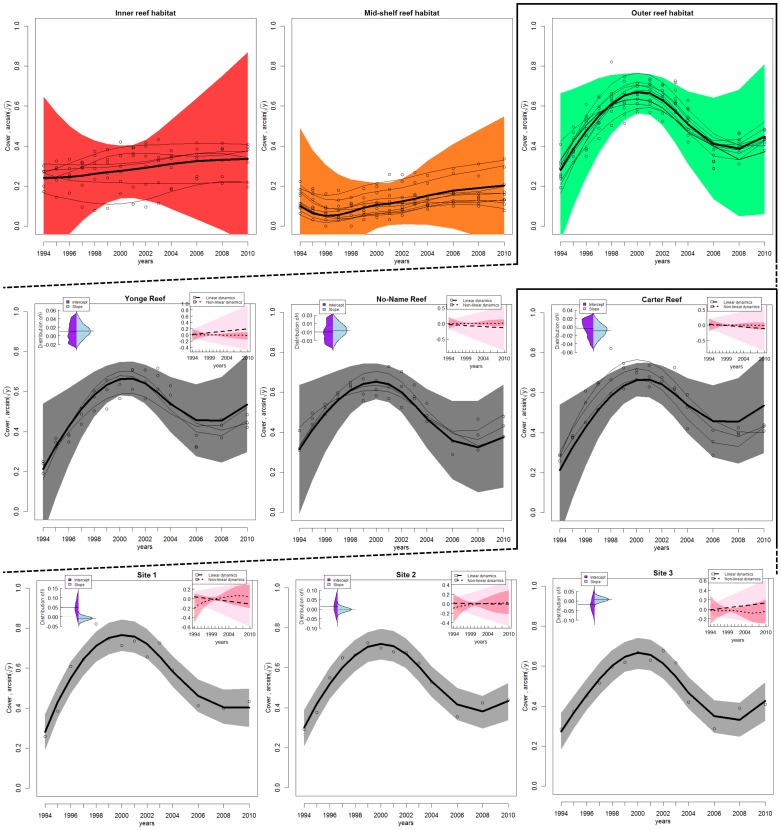
Observed (dots) and fitted (curves) *Acropora* dynamics and associated parameter estimates and uncertainties. Coral dynamics are modelled at the three spatial scales within the sub-region of Cooktown-Lizard Island; with plotted lines for: habitats (top); reefs (middle); and sites (bottom). Shaded areas encompass 95% posterior predictive intervals around estimated coral trajectories and 95% credible intervals around model parameters. Top-right inserts on plots illustrate the linear and non-linear components of coral trajectories extracted from equations for *f_h_*, *f_r_* and *f_i_*. Top-left inserts illustrate posterior distributions of linear parameters (from the top to the bottom, plotted lines are for: *θ_0_* and *θ_1_*, *δ_0_* and *δ_1_* respectively; refer to Fig. 2, see equations in main text). Intercept terms were indexed by 0 and slope terms by 1 and shown with their 95% credible interval. Thin black lines on reef- and habitat-scale plots (mid- and top-line) show the fitted dynamics of nested individual sites. Note different y-axis scales in inserted graphs. Estimates of coral cover trajectories are illustrated at the three sites at Carter Reef from the outer reef habitat depicted in Figure 1.

**Figure 4:**
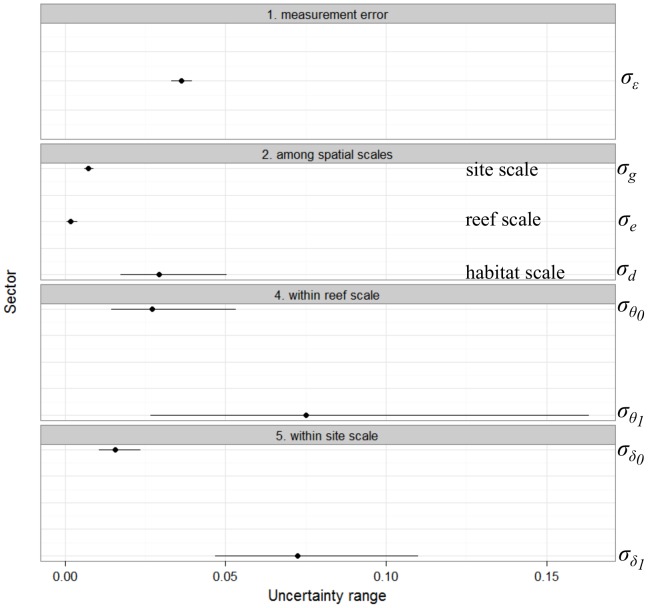
Estimation of the uncertainty in the three hierarchical stages of the model. The measurement error *σ_ε_* corresponds to the uncertainty at the data stage, and all other variance components *σ* account for uncertainty in the process model, decomposed into the variability in trend (intercept and slope) of coral cover at the three spatial scales of habitat, reefs and sites.

Visualization of posterior predictions compared to observations indicated that the model successfully captured spatial and temporal variability in *Acropora* dynamics at the three spatial scales nested within sub-region (Fig. 3). However, posterior distributions of the slope and intercept in trend (i.e, fixed component) show that their precision decreased with the scale of observation (see top-left inserts on plots in Fig. 3, Fig. S1). At the finer scale of the site, linear components of the trend were relatively tightly estimated: slopes were centred around 0 (range of 95% credible interval, RCI<0.05) and intercept terms had slightly larger variances (RCI≈0.1) with occasional non-zero central values. At the intermediate scale of the reef, the variance of the trend parameters was also relatively small (RCI<0.1). At the broadest scale, however, the estimated slopes and intercepts in trend for all three habitats were poorly estimated (Fig. S1). These results demonstrate that the temporal trends in the model successfully capture patterns at the site and reef scales, but not at the broader scale of habitat. At the finer spatial scales, linear dynamics were different between and within reefs and sites, with a consistent increase in the amount of uncertainty in more recent years (top-right inserts on plots in Fig. 3). The estimated non-linear dynamics were consistent within each spatial scale with spline contributions close to zero, but the degree of uncertainty differed between scales and was smallest at the reef scale, intermediate at the site, and largest at the habitat scale (top-right insert on each plot in Fig. 3). As expected, these uncertainties increased when moving from the centre to the edges of the surveyed period. The relatively narrow range of the measurement error (RCI≈0.01, Fig. 4) illustrates that coral dynamics were well explained by the multi-scale dynamics model given the data, model structure and distributional assumptions.

### Modelled coral trajectory patterns

Most of the uncertainty in estimated coral trajectories occurred among habitats (Fig. 4). Indeed, *Acropora* dynamics were substantially different at this scale of observation. Overall coral cover was least on mid-shelf reefs, intermediate in inner habitats, and largest on outer reefs (Fig. 3). Inner- and mid-shelf reefs showed a slow increase in coral cover over the 14 years of survey, with some evidence of previous decline in the mid-shelf habitat. Outer reefs showed more pronounced trajectories, with a rapid increase in coral cover between 1994 and 2000, a sharp decline from 2000 to 2008, and early signs of recovery afterward.

Within the sub-region of Cooktown-Lizard Island, *Acropora* population trajectories were relatively consistent among sites and reefs with narrow uncertainty in model parameters estimation (RCI<0.01), however, the degree of uncertainty was higher within reefs and sites particularly in the estimation of variance components for slope in the trend,
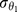
and
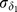
(Fig. 4). These results indicate that estimation of coral cover is affected by diverse sources of uncertainty acting at different stages of the model and levels of observation.

## Discussion

Mathematical description of non-linear trends and associated uncertainties for hierarchically structured data over different spatial scales is a very big challenge which is extremely difficult to address using existing statistical approaches. This limitation severely impacts our abilities to understand a diverse range of ecological systems, and other systems more broadly. For example, long-term surveys of ecosystems are typically based on hierarchical observations [3], [8]; in epidemiology, spatial trends in health outcomes are monitored among different hierarchies in populations; in finance, temporal patterns in returns are monitored among financial sectors; and so on. In this paper, we introduce a new statistical approach that explicitly accounts for variability and uncertainty present in population trajectory models by coupling hierarchical and semi-parametric methods. Hierarchical modelling by its very nature allows the partitioning of variability into multiple spatial scales and model stages. It has been well demonstrated that ecological processes can be highly variable both within and across different spatio-temporal scales and that the relationship between two variables can change according to the spatial scale considered [8], [51]. As a consequence, robust quantitative descriptions of natural patterns need to be able to connect broad-scale patterns to fine-scale processes, and in doing so transfer information across these scales [52]. Complemented by a semi-parametric formulation, our approach here facilitates a more flexible representation of trends over time, free of parametric constraints, and thereby, more effectively “letting the data speak for themselves” [7]. In addition, we modelled multifaceted trajectories of populations across space and time by decomposing dynamics into linear and non-linear components which represent deviations from linearity. Thus, we are able to simultaneously identify long-term trends (linear) as well as more temporally localized variations (often non-linear) in population trajectories [12], contributing to better diagnosis of sources of variability inherent in long-term ecological data.

There is a growing literature on the philosophical, practical and inferential benefits of the Bayesian framework for modelling ecological data [2], [3], [6], [31], [38], [53]. In the field of coral reef ecology, applications of Bayesian hierarchical regression models are fairly new. Published examples have examined: the effects of temperature anomalies on coral cover declines from a global meta-analysis [17] and spatial variability of reef fish community structure in French Polynesia [9] and Australia [26]. These studies, however, investigated the effects of different covariates without full consideration of the associated uncertainties in their models. As a consequence, these studies have not fully utilized a major benefit of Bayesian computation, which resides in its capacity to simultaneously quantify uncertainties associated with estimated data, model structure and model parameters. Indeed, we are no longer restricted to making a choice: whether to account for the spatio-temporal structure in the data whilst only tracking positive or negative effects of covariates on a response variable; or to simplify the spatial structure whilst tracking non-linear effects of the covariates on the response. By accounting for non-linear trends (in the fixed component of the model), we may then focus on the noise at the appropriate spatio-temporal scales (in the random effects component). For coral reef trajectories, we must allow sufficient complexity in both of these fixed and random components. Doing so will help identify and potentially reduce sources of uncertainty associated with modelling and will contribute to improved knowledge of the dynamics of populations studied in this way.

The Bayesian semi-parametric hierarchical model presented here also provides a more ecologically relevant way of modelling population trajectories, in cases where variability is large and cannot be assigned to unique causes and origins. By decomposing this variation into variance components at multiple spatial scales and model stages, and directly assessing the posterior distributions of these components, we have shown that it is not possible to accurately estimate trend parameters at a spatial scale larger than that of a reef in the sub-region considered here. That is, coral trajectories were consistent at the km^2^ scale of sites within reefs but diverged at the larger 5 km^2^ scale of reefs within habitats. These results suggest that time as a single covariate is insufficient to explain the coral trajectories at the habitat or sub-regional scales, and would benefit from additional explanatory factors, such as processes operating at these larger scales. For example, a historical review of *Acropora* populations within the Cooktown-Lizard Island sub-region modelled here reveals that they were affected by outbreaks of the coral predator crows-of-thorns starfish (*Acanthaster planci*) on mid-shelf reefs between 1995 and 1999, and by white syndrome disease from 2000 to 2003 on outer reefs [12]. Crowns-of-thorns starfish outbreaks are relatively slow and diffusive disturbances whose propagation is driven by prey availability [16]. Similarly, the spread of diseases and impacts from other major disturbances such as cyclones and coral bleaching are not homogenous on reefs and typically attenuate as the result of multiple factors acting at differing scales [12], [28]. As a consequence, the effects of disturbances are seldom homogeneous across reefs, particularly at larger spatial scales such as, in the case of the GBR, shelf-position, making it difficult to explain coral trajectories using time as a single covariate. Nonetheless, this sort of approach is sometimes adopted [12]–[14]. Moreover, the three categories of shelf-position sampled by the LTMP were initially defined for management purposes but in a largely *ad hoc* fashion and were based on limited knowledge of reef ecology at that time [21]. Indeed, the observed reduction in the precision of parameter estimates at larger spatial scales can be partly attributed to a lack of trends in the data at this habitat scale. A remedy for this may be to include informative covariates in the model or use more informed prior information [54], obtained perhaps from similar analyses of other coral reef systems; these options are the subject of ongoing research. Moreover, based on the more extensive information extracted from our statistical approach, more informative spatial sampling programs could be designed to address specific management issues in an adaptive learning framework as advocated for example by [55]. At present, however, our results indicate that in the absence of better prior knowledge, other explanatory covariates and/or other spatial designs, conclusions about GBR coral cover trajectories become more uncertain at a scale larger than individual reefs. Therefore, in this context, management actions and the assessment of their efficacy may be better focused at the reef scale.

In conclusion, the Bayesian semi-parametric hierarchical approach introduced here facilitates flexible and environmentally relevant description of non-linear population trajectories and associated uncertainties. As illustrated, it can be used to identify critical spatial thresholds beyond which ecological data reveal divergence in the trajectories and so hinder model efficiency. Without this decomposition of uncertainties at multiple spatial scales and model stages, patterns remain concealed and conclusions regarding population trajectories can be considerably compromised. In contrast, we argue that our model can unlock information contained in spatially extensive time-series data, facilitate the design of better future surveys, provide guidance for understanding sources of uncertainty, and support better informed decision making.

## Supporting Information

Figure S1 **Illustration of the poor estimation of slope (** ***γ_1h_*** **) and intercept (** ***γ_0h_*** **) parameters based on three MCMC chains from a simulation of 200,000 iterations, a burn-in of 100,000 iterations and a thinning rate of 50 iterations.** Differences in estimated values in MCMC chains demonstrate the non-convergence of the algorithm to a unique posterior distribution for each parameter. This non-convergence also affects the variance of the parameters with ranges of the 95% credible intervals (RCIs) varying between 0.1 and 1.
(TIFF)

Code S1 **R and WinBUGS code to implement the Bayesian semi-parametric hierarchical model.**
(DOCX)
