# Supplementary figures and images for: Understanding Uncertainties in Non-Linear Population Trajectories: A Bayesian Semi-Parametric Hierarchical Approach to Large-Scale Surveys of Coral Cover
Source: PLoS One. 2014 Nov 3;9(11):e110968. doi: 10.1371/journal.pone.0110968 (PMC4217738; doi:10.1371/journal.pone.0110968)

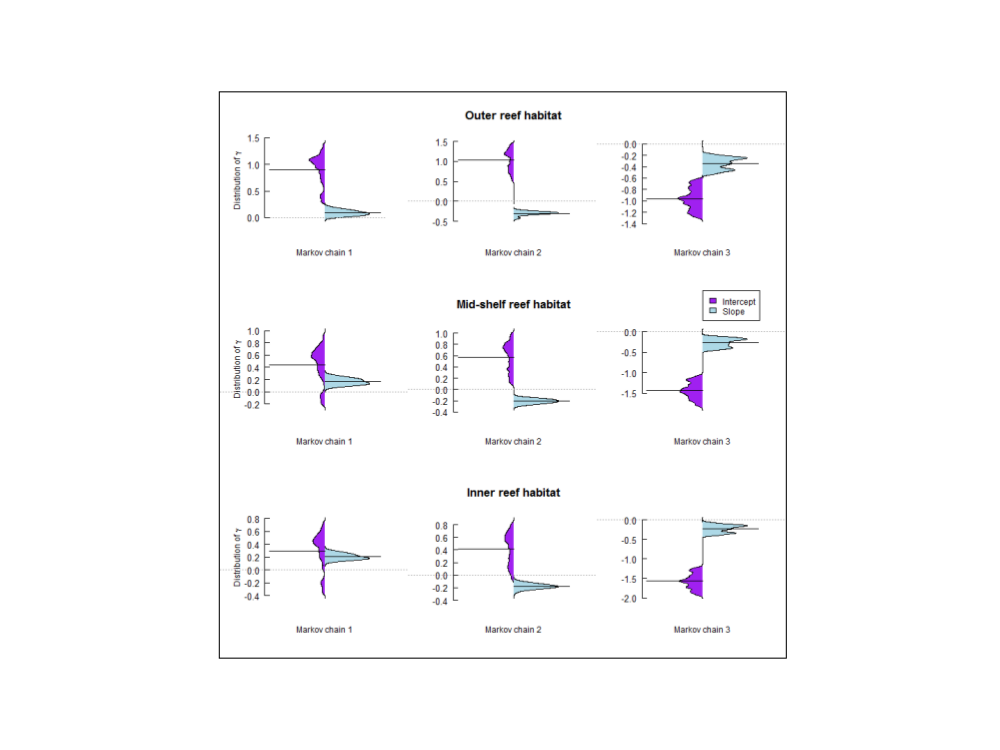

Supplement: Figure S1 — Illustration of the poor estimation of slope ( γ1h ) and intercept ( γ0h ) parameters based on three MCMC chains from a simulation of 200,000 iterations, a burn-in of 100,000 iterations and a thinning rate of 50 iterations. Differences in estimated values in MCMC chains demonstrate the non-convergence of the algorithm to a unique posterior distribution for each parameter. This non-convergence also affects the variance of the parameters with ranges of the 95% credible intervals (RCIs) varying between 0.1 and 1. (TIFF) [file pone.0110968.s001.tiff]
