# Supplementary material for: Understanding Uncertainties in Non-Linear Population Trajectories: A Bayesian Semi-Parametric Hierarchical Approach to Large-Scale Surveys of Coral Cover
Source: PLoS One. 2014 Nov 3;9(11):e110968. doi: 10.1371/journal.pone.0110968 (PMC4217738; doi:10.1371/journal.pone.0110968)
Supplement: Code S1 — R and WinBUGS code to implement the Bayesian semi-parametric hierarchical model. (DOCX) [file pone.0110968.s002.docx]

**Supporting Information**

#################### BAYESIAN SEMI-PARAMETRIC HIERARCHICAL MODEL ####################

# Estimation of long-term trajectory of *Acropora* cover from the Great Barrier Reef using four spatial scales: sub-region, habitat, reef and site.

# see Vercelloni et al 2014 for further details.

# Load in required packages:

library(lattice); library(coda); library(R2WinBUGS)

setwd("C:\\Julie\\WinBUGS_model")

# Read the data

Coral.df<-read.csv(file.choose())

RecCL<- Coral.df[Coral.df$Sector=="CL",] # choose of the sub-region

numObs <- length(RecCL$asinmeanCC) # total number of observations

### Create site variable

idnumOrig <- RecCL$site

idnum <- rep(NA,length(idnumOrig))

uqID <- unique(idnumOrig)

for (i in 1:length(uqID))

idnum[idnumOrig==uqID[i]] <- i

RecCL$idnum <- idnum

numSubj <- length(unique(idnum)) ### Indivivdual number

uqID <- unique(idnum) # Individual patch name

### Create reef variable

idreeOrig <- RecCL$Reefnumber

idree <- rep(NA,length(idreeOrig))

uqree <- unique(idreeOrig)

for (i in 1:length(uqree))

idree[idreeOrig==uqree[i]] <- i

RecCL$idree <- idree

numree <- length(unique(idree)) #Reef number

uqree <- unique(idree) # Reef name

### Create habitat variable

idgrpOrig <- RecCL$ShelfPosition

idgrp <- rep(NA,length(idgrpOrig))

uqGP <- unique(idgrpOrig)

for (i in 1:length(uqGP))

idgrp[idgrpOrig==uqGP[i]] <- i

RecCL$idgrp <- idgrp

numGrp <- length(unique(idgrp)) # Habitat number

uqGP <- unique(idgrp) # Habitat name

### Centred years around the middle of the survey

years<-RecCL$Years

y<-RecCL$Years[1:14]

ybarre<-mean(y)

yearsCent<-(y-ybarre)

for (h in 1:nrow(RecCL)){

for (d in 1:14){

if (RecCL$Years[h]==years[d]){

RecCL$yearsCent[h]<-yearsCent[d]

}}}

### Creation matrix X: fixed coefficients matrix (Crainiceanu et al. 2005)

yearsCent<-RecCL$yearsCent

Yn<-length(yearsCent)

num.knots=4

X<-cbind(rep(1,Yn),yearsCent)

knots<-quantile(unique(yearsCent),

seq(0,1,length=(num.knots+2))[-c(1,(num.knots+2))])

### Design matrix of random coefficients Z (Crainiceanu et al. 2005)

Z_K<- (abs(outer(yearsCent,knots,"-")))^3

OMEGA_all<-(abs(outer(knots,knots,"-")))^3

svd.OMEGA_all<-svd(OMEGA_all)

sqrt.OMEGA_all<-t(svd.OMEGA_all$v %*%

t(svd.OMEGA_all$u)*sqrt(svd.OMEGA_all$d))

Z<-t(solve(sqrt.OMEGA_all,t(Z_K)))

### Implentation model

program.file.name="BayesianSemiParametricHierarchicalModel.txt" # WinBUGS model

init.C<-rep(0.01,num.knots)

init.D<-(array(0.01,c(numGrp,num.knots)))

init.E<-(matrix(0.01,numree,num.knots))

init.G<-(matrix(0.01,numSubj,num.knots))

init.Gam<-matrix(0.01,numGrp,2)

init.Te<-matrix(0.01,numree,2)

init.De<-matrix(0.01,numSubj,2)

response <- RecCL$asinmeanCC

n <-numObs

nsites <-numSubj

ngroups <-numGrp

nreef <-numree

group<-RecCL$idgrp

reef<-RecCL$idree

site<-RecCL$idnum

data<-list("response","X","Z","n","nsites","nreef","ngroups","num.knots","group","reef","site")

inits<-function(){list(beta=c(0,0),c=init.C,tauc=1,d=init.D,taud=1,e=init.E,taue=1,g=init.G,

taug=1,taueps=1,gamma=init.Gam,teta=init.Te,

delta=init.De,taudelta=c(1,1),tauteta=c(1,1))}

parameters<-list("mean","freef","fgroup","beta","delta","gamma","teta",

"splinei","spliner","splineh","taueps","fsector","splines",

"tauc","taud","taue","taug","taudelta","tauteta")

# Model

Bayes.fit<-bugs(data,inits,parameters,model.file=program.file.name, n.chains=3, bugs.directory = "C:\\Julie\\WinBUGS14",DIC=TRUE, n.iter=200000,n.burnin=100000,n.thin=50, debug=T)

attach.all(Bayes.fit)

**WinBUGS model** (save in .txt)**:**

model{

#Likelihood of the model

for (k in 1:n) {

response[k]~dnorm(mean[k],taueps)

# Assess model fit using a sum-of-squares-type discrepancy

residual[k] <- response[k]-mean[k] # Residuals for observed data

sq[k] <- pow(residual[k], 2) # Squared residuals

}

# Calcul of expected values

for (k in 1:n) {

mean[k]<-f[k]+fg[k]+fr[k]+fi[k]

f[k]<-beta[1]*X[k,1]+beta[2]*X[k,2]+splines[k]

fg[k]<-gamma[group[k],1]*X[k,1]+gamma[group[k],2]*X[k,2]+splineh[k]

fr[k]<-teta[reef[k],1]*X[k,1]+teta[reef[k],2]*X[k,2]+spliner[k]

fi[k]<-delta[dog[k],1]*X[k,1]+delta[dog[k],2]*X[k,2]+splinei[k]

splines[k]<-b[1]*Z[k,1]+b[2]*Z[k,2]+b[3]*Z[k,3]

splineh[k]<-c[group[k],1]*Z[k,1]+c[group[k],2]*Z[k,2]+c[group[k],3]*Z[k,3]

spliner[k]<-w[reef[k],1]*Z[k,1]+w[reef[k],2]*Z[k,2]+w[reef[k],3]*Z[k,3]

splinei[k]<-d[dog[k],1]*Z[k,1]+d[dog[k],2]*Z[k,2]+d[dog[k],3]*Z[k,3]

}

#Prior for the random parameters of the sub-region curve

for (m in 1:num.knots){

c[m]~dnorm(0,tauc)}

#Prior for the random parameters for the curves describing habitats

for (m in 1:num.knots){

for (n in 1:ngroups){

d[n,m]~dnorm(0,taud)}}

#Prior for the random parameters for the curves describing reefs

for (k in 1:num.knots){

for (h in 1:nreef){

e[h,k]~dnorm(0,taue)}}

#Prior for the random parameters for sites

for (i in 1:nsites){

for (m in 1:num.knots){

g[i,m]~dnorm(0,taug)}}

#Prior for monomial parameters of the sub-region

for (l in 1:2){

beta[l]~dnorm(0,0.001)}

#Prior for monomial parameters of curves describing the habitats

for (l in 1:2){

for (j in 1:ngroups){

gamma[j,l]~dnorm(0,0.001)}}

#Prior for monomial parameters of curves describing the reef

for (l in 1:2){

for (h in 1:nreef){

teta[h,l]~dnorm(0,tauteta[l])}}

#Prior for monomial parameters of curves describing sites

for (i in 1:nsites){

for (l in 1:2){

delta[i,l]~dnorm(0,taudelta[l])}}

#Priors of precision parameters

tauc~dgamma(1.0E-3,1.0E-3)

taud~dgamma(1.0E-3,1.0E-3)

taue~dgamma(1.0E-6,1.0E-6)

taug~dgamma(1.0E-3,1.0E-3)

taueps~dgamma(1.0E-3,1.0E-3)

for (l in 1:2){

taudelta[l]~dgamma(0.001,0.001)

tauteta[l]~dgamma(0.001,0.001)}

#Define the reef, habitat and sub-region curves

for (k in 1:n){

freef[k]<-f[k]+fg[k]+fr[k]

fgroup[k]<- f[k]+fg[k]

fsector[k]<-f[k]}

}

#End model
